# Hearing Loss in Adults With Diabetes and Prediabetes: A Systematic Review and Meta‐Analysis

**DOI:** 10.1002/dmrr.70195

**Published:** 2026-06-23

**Authors:** Mehwish Nisar, Shamshad Karatela, Anjana Rajagopal, Beenish Nisar Ahmed, Piers Dawes

**Affiliations:** ^1^ Centre for Hearing Research, School of Health and Rehabilitation Sciences The University of Queensland Brisbane Australia; ^2^ School of Public Health and Preventive Medicine Monash University Melbourne Australia; ^3^ School of Health and Rehabilitation Sciences The University of Queensland Brisbane Australia; ^4^ Department of Otorhinolaryngology and Head & Neck Surgery Avicenna Medical Complex Hospital Islamabad Pakistan; ^5^ College of Medicine Bahria University Islamabad Pakistan

**Keywords:** audiology, moderate‐to‐severe hearing impairment, risk assessments, screening, type 2 diabetes

## Abstract

Diabetes impairs hearing through microvascular damage and neuropathy, yet the prevalence of moderate‐to‐severe hearing loss (≥ 40 dB HL) remains inadequately explored. Variations by age, diabetes duration, and socioeconomic factors are inadequately characterised. This systematic review quantified the prevalence and comparative risk of moderate‐to‐severe hearing loss in diabetes and prediabetes, exploring variations across age, national income level, and disease duration. We searched PubMed, Scopus, Web of Science, SPORTDiscus, and CINAHL (2000–2025) for observational studies reporting audiometric thresholds in diabetic or prediabetic subjects (PROSPERO: CRD42018100742). Quality was assessed using the Newcastle–Ottawa Scale. Random‐effects meta‐analyses generated pooled prevalence and odds ratios (ORs) with 95% confidence intervals (CIs). Publication bias was evaluated via funnel plots and Egger’s regression. Of 3490 records, 29 studies qualified. Most examined type 2 diabetes; one included prediabetes. Twenty‐three studies (*n* = 5221) yielded a pooled prevalence of 24% (95% CI: 19%–30%; *I* ^2^ = 94%). Eleven studies showed diabetes doubled hearing loss odds versus controls (OR = 2.41, 95% CI: 1.62–3.60; *I* ^2^ = 86.6%). Risk was significantly elevated in younger adults (< 60 years: OR = 3.03, 95% CI: 2.17–4.22) but not in older adults (≥ 60 years: OR = 1.52, 95% CI: 0.72–3.22). Low‐ and middle‐income countries showed the highest risk (OR = 4.51, 95% CI: 2.43–8.40) versus high‐income countries (OR = 1.78, 95% CI: 1.05–3.02). Diabetes duration < 10 years conferred elevated risk (OR = 2.68). Small‐study effects were detected (Egger’s *p* = 0.019) but sensitivity analyses confirmed robustness. One in four diabetic adults has clinically significant hearing loss, particularly in younger individuals and resource‐limited populations. These findings support the integration of routine audiometric screening into diabetes care.

## 1 Introduction

Diabetes mellitus (DM) represents an escalating global health crisis, currently impacting over 537 million adults worldwide, with projections indicating a rise to 783 million by 2045 [1]. While retinopathy, nephropathy, and neuropathy are conventionally recognised and routinely monitored microvascular complications of diabetes, hearing impairment has emerged as a significant, yet frequently overlooked, sensory complication [2]. Compelling evidence suggests that chronic hyperglycaemia causes damage to the microvasculature and neural pathways within the cochlea. This damage, mediated by mechanisms such as microangiopathy, oxidative stress, and neuropathy, ultimately culminates in sensorineural hearing loss [3]. On average, individuals with diabetes experience nearly double the incidence of hearing loss compared with non‐diabetic individuals, although evidence for prediabetes remains limited and inconsistent [4]. Despite these clear pathophysiological links, hearing loss is not yet systematically integrated into diabetes care protocols, leading to an incompletely characterised true burden [4].

Hearing impairment is far from a benign sensory deficit; its effects are profound, impacting communication, social engagement, mental health, and increasing listening effort [5]. The detrimental impacts of hearing loss become especially pronounced at moderate‐to‐severe levels (defined as audiometric hearing thresholds ≥ 40 dB hearing level [HL]), where conversational understanding is substantially compromised [6]. Individuals with moderate‐to‐severe hearing loss struggle to follow speech in noisy environments and often require hearing aids or other assistive devices [7]. Hearing loss progresses gradually, with patients remaining unaware of their auditory decline until functional limitations become evident [8]. Crucially, clinically significant hearing loss is detectable through straightforward, low‐cost audiometric screening, presenting a vital opportunity for early diagnosis and intervention [9]. For individuals with diabetes already navigating complex disease management, disabling hearing loss represents a compounding challenge that further erodes the quality of life and impairs self‐care behaviours [9, 10].

Despite the clear clinical and public health implications of diabetes‐associated hearing loss, the existing epidemiological evidence remains fragmented and inconsistent [10]. Previous systematic reviews and meta‐analyses, while establishing a connection, have largely encompassed hearing impairment of any severity [11, 12]. These often include mild or subclinical deficits that, despite audiometric detectability, hold limited functional relevance for daily communication and typically do not warrant immediate clinical intervention. While meta‐analyses indicate that individuals with diabetes have approximately twice the odds of hearing loss (OR = 2.15; 95% CI: 1.72–2.68) [11], with recent reviews of Type 2 Diabetes reporting similar elevations [13, 14], their inclusion of all severity levels may overstate the clinically actionable burden. These analyses also highlight higher mean audiometric thresholds in diabetic individuals and an association between longer diabetes duration and poorer glycaemic control with greater hearing loss prevalence [13, 14].

However, by largely aggregating all hearing loss severity levels, prior syntheses have inadvertently obscured the true scope of functionally disabling hearing loss. Consequently, critical knowledge gaps persist. Foremost, no meta‐analysis has specifically quantified the burden of moderate‐to‐severe hearing loss (≥ 40 dB HL), a crucial omission given this threshold signifies auditory dysfunction warranting clinical intervention, with its pooled prevalence and risk uncharacterised [15]. Furthermore, the modifying influence of age is insufficiently understood; presbycusis may obscure effects in older adults, while younger adults offer clearer insights into metabolic mechanisms [16, 17]. The socioeconomic context is also largely unexplored: burden in LMICs may be disproportionately high due to suboptimal care, potentially attenuated in HICs [18]. Additionally, diabetes duration warrants specific exploration for clinically significant hearing loss, as prolonged hyperglycaemia contributes to cumulative cochlear damage [19].

To address these critical gaps comprehensively, we conducted a systematic review and meta‐analysis focussing exclusively on screen‐detected, clinically significant hearing loss (≥ 40 dB HL) among adults with diabetes and prediabetes. The aims of this study were to: (1) estimate the pooled prevalence of moderate‐to‐severe hearing loss in diabetic populations; (2) quantify the comparative risk versus normoglycemic controls; and (3) examine effect modification by age, diabetes duration, and national income level where sufficient data were available. By focussing on clinically meaningful thresholds and systematically exploring heterogeneity sources, this review provides precise, policy‐relevant estimates of the diabetes‐related hearing impairment burden. Ultimately, these findings support integrating routine audiometric screening into diabetes care pathways, prioritising resource‐limited settings and younger populations where early intervention yields the greatest benefit.

## 2 Methods

### 2.1 Protocol Registration and Reporting Standards

This systematic review and meta‐analysis were prospectively registered with the International Prospective Register of Systematic Reviews (PROSPERO; registration number: CRD42018100742) and conducted in accordance with the Preferred Reporting Items for Systematic Reviews and Meta‐Analyses (PRISMA) guidelines to ensure methodological transparency and reproducibility [20]. Complete PRISMA compliance was verified through systematic application of both the main 27‐item reporting checklist (Supporting Information S1) and the abstract‐specific 12‐item checklist (Supporting Information S2).

### 2.2 Search Strategy and Information Sources

A comprehensive systematic literature search was conducted across five electronic databases: PubMed, Scopus, Web of Science, SPORTDiscus, and CINAHL, covering the period from January 2000 to August 2025. These databases were selected for their extensive coverage of health sciences literature and multidisciplinary research. To minimise publication bias, grey literature searches were performed using Google Scholar, ProQuest Dissertations and Theses, and websites of relevant professional organisations, including diabetes associations, audiology societies, and public health agencies. Additionally, reference lists of all included studies underwent manual screening to identify potentially eligible sources not captured through database searches. The search strategy was developed and refined in consultation with a qualified medical research librarian to optimise sensitivity and specificity (Supporting Information S3).

### 2.3 Study Selection Process

Retrieved articles underwent a two‐stage screening process. Initially, titles and abstracts were assessed by two independent reviewers (M.N. and S.K.) to identify potentially eligible studies. Subsequently, full‐text articles of potentially relevant studies were independently evaluated by two reviewers (M.N. and A.R.) against predefined eligibility criteria. Any discrepancies between reviewers were resolved through discussion, with a third independent reviewer (B.N.A.) consulted when consensus could not be reached.

### 2.4 Eligibility Criteria

Studies were included if they met the following criteria: (1) peer‐reviewed original research published in English; (2) observational study designs (cohort, case‐control, or cross‐sectional); (3) adult participants (≥ 18 years) with clinically diagnosed diabetes or prediabetes; (4) hearing assessment conducted using objective audiometric methods (like, pure‐tone audiometry); (5) reporting of hearing loss prevalence data, particularly moderate‐to‐severe impairment (≥ 40 dB HL); and (6) publication between January 2000 and 31st August 2025.

Studies were excluded if they: (1) reported only incidence data rather than prevalence; (2) contained duplicate datasets already included from other publications; (3) focused on gestational diabetes, occupational noise exposure, ototoxic medication use, or known ear pathologies such as chronic otitis media or conductive hearing loss, as these could confound the association between diabetes and sensorineural hearing impairment; or (4) relied solely on self‐reported hearing status without objective audiometric confirmation.

### 2.5 Data Extraction

Data extraction was performed independently by two reviewers (M.N. and A.R.) using a standardised, piloted data extraction template to ensure consistency and completeness. Discrepancies were resolved through discussion, with unresolved conflicts adjudicated by a third reviewer. Extracted variables included: publication year, study location, sample size, participant age (mean or range), sex distribution (proportion female), study design, recruitment setting (hospital‐based, community‐based, or population‐based), diabetes type (type 1, type 2, or mixed), diabetes duration, hearing assessment methodology, hearing loss threshold definitions, and prevalence estimates for moderate‐to‐severe hearing loss (≥ 40 dB HL).

### 2.6 Quality Assessment

Methodological quality of the included studies was independently assessed by two reviewers (M.N. and A.R.) using the Newcastle‐Ottawa Scale (NOS), a validated tool for evaluating non‐randomised studies [21]. The NOS assigns scores ranging from 0 to 9 based on three domains: selection of study groups, comparability of groups, and ascertainment of exposure/outcome. Studies were categorised as poor quality (0–2), fair quality (3–5), or good/high quality (6–9). Quality scores were used for sensitivity analyses but did not serve as exclusion criteria [22].

### 2.7 Statistical Analysis

Random‐effects meta‐analyses were conducted using the meta package in R Studio to generate pooled prevalence estimates [23] of moderate‐to‐severe hearing loss (≥ 40 dB HL) with 95% confidence intervals (CIs). Pooled odds ratios (ORs) quantified the association between diabetes and hearing impairment relative to non‐diabetic controls. Between‐study heterogeneity was assessed using the *I* ^2^ statistic (25%, 50%, 75% indicating low, moderate, high heterogeneity, respectively). Subgroup analyses explored effect modification by: (1) mean age (< 60 vs. ≥ 60 years) to account for presbycusis [16], (2) national income level (LMICs vs. HICs, per World Bank classifications), examining socioeconomic disparities [24] and (3) diabetes duration (< 10 vs. ≥ 10 years), assessing dose‐response relationships where data permitted. We conducted pre‐specified subgroup analyses stratified by age, national income level, and diabetes duration to investigate heterogeneity sources. Given the limited number of included studies (*n* = 11), we employed this approach rather than multivariable meta‐regression, which would provide insufficient statistical power and risk overfitting, consistent with Cochrane recommendations for meta‐analyses with limited study numbers [25]. Publication bias was evaluated via funnel plot inspection and Egger’s regression test (*p* < 0.10 indicating small‐study effects). Sensitivity analyses assessed robustness by sequentially excluding lower‐quality studies and evaluating individual study influence on pooled estimates [23].

## 3 Results

### 3.1 Search Results

**FIGURE 1:**
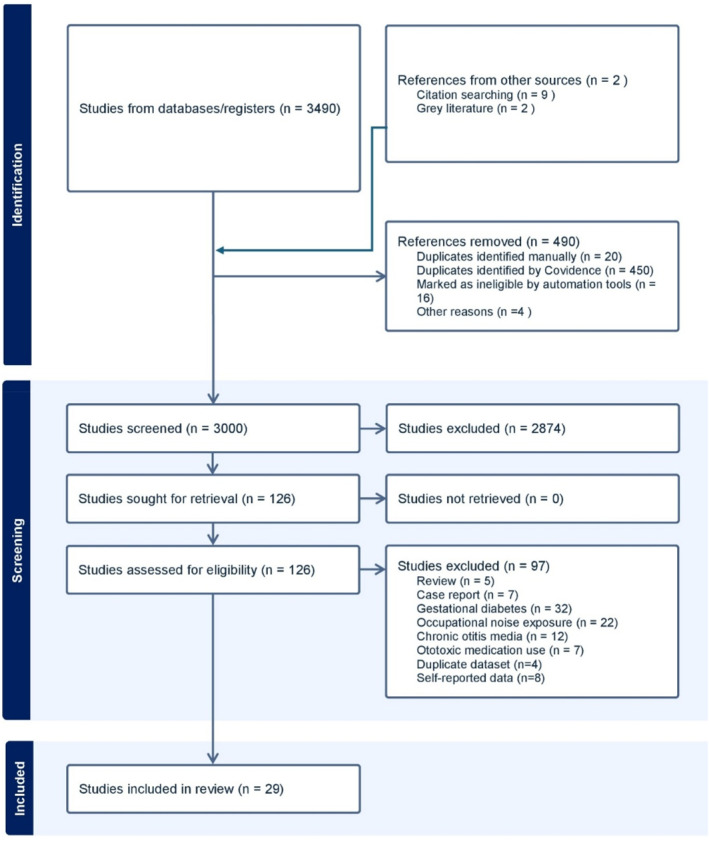
PRISMA flow diagram of study selection process.

The database search identified 3490 records from PubMed, Scopus, Web of Science, SPORTDiscus, and CINAHL between January 2000 and 31st August 2025. After removal of duplicates and screening of titles and abstracts, 126 articles underwent full‐text review. Of these, 29 studies met the inclusion criteria [26, 27, 28, 29, 30, 31, 32, 33, 34, 35, 36, 37, 38, 39, 40, 41, 42, 43, 44, 45, 46, 47, 48, 49, 50, 51, 52, 53, 54] and were included in the results synthesis, and 27 studies of fair‐to‐good quality contributed data to the quantitative meta‐analysis [26, 27, 28, 29, 30, 31, 32, 33, 34, 35, 36, 37, 38, 39, 40, 41, 42, 43, 44, 45, 46, 47, 48, 49, 50, 51, 52]. The selection process is summarised in the PRISMA flow diagram (Figure 1). Most of the studies were excluded from non‐audiometric or self‐reported hearing data, gestational diabetes, or overlapping populations.

### 3.2 Characteristics of Included Studies

The detailed characteristics of the included studies are provided in Table 1. Included studies were conducted across Asia (*n* = 14), Africa (*n* = 4), the Middle East (*n* = 3), Europe (*n* = 3), North America (*n* = 3), and Australia (*n* = 2), representing a broad geographic and socioeconomic distribution [26, 27, 28, 29, 30, 31, 32, 33, 34, 35, 36, 37, 38, 39, 40, 41, 42, 43, 44, 45, 46, 47, 48, 49, 50, 51, 52, 53, 54]. Study designs comprised cross‐sectional (*n* = 18), case–control (*n* = 7), and cohort (*n* = 2) studies [26, 27, 28, 29, 30, 31, 32, 33, 34, 35, 36, 37, 38, 39, 40, 41, 42, 43, 44, 45, 46, 47, 48, 49, 50, 51, 52, 53, 54]. The majority of participants had type 2 diabetes, with one study including individuals with prediabetes and a few reporting mixed diabetes types [35]. Participants were adults aged 18–86 years, with a roughly balanced sex distribution (40%–60% female; Table 1). Screening settings were hospital/clinic in 69%, community programmes in 21%, and national survey samples in 10% of included studies [26, 27, 28, 29, 30, 31, 32, 33, 34, 35, 36, 37, 38, 39, 40, 41, 42, 43, 44, 45, 46, 47, 48, 49, 50, 51, 52, 53, 54]. Hearing status was assessed almost universally by pure‐tone audiometry, supplemented in some studies by tympanometry, distortion product otoacoustic emissions (DPOAEs), or auditory brainstem response (ABR) testing [55]. Moderate‐to‐severe hearing loss was consistently defined as hearing threshold ≥ 40 dB HL in the better ear, in line with World Health Organisation criteria [56]. Studies varied in whether thresholds were calculated for speech frequencies (0.5–4 kHz) or full audiometric ranges. Quality assessment (Supporting Information S4) using the Newcastle–Ottawa Scale indicated that 18 studies were of good quality (6–9 stars), seven were fair (3–5 stars), and two were poor (< 3 stars). No high risk of selection bias was identified, though several cross‐sectional studies lacked adjustment for potential confounders such as noise exposure and ototoxic drug use.

**TABLE 1:** Study characteristics of included studies.

Authors (year)	Country	Study design	Screening setting	Screening dates	Population (*N*, mean age, %female)	Diabetes type	Hearing assessment	*N* screened	Hearing loss metrics—cases	Hearing loss metrics—controls	Prevalence of hearing loss (cases)	Associated factors
Adebola et al. 2016 [26]	Nigeria	Cross‐sectional, comparative	Hospital	December 2013–May 2014 (6 months)	*N* = 187 (DM 97; control 90); 58.9 & 58.8 years; 55%–56% F	Type 2	Otoscopic exam, pneumatic otoscopy, PTA	187	Abnormal PTA 21.5%; SNHL 61.9%; mixed 23.9%; mild 19%; mod 38%; Sev 24%; Prof 19%	Abnormal PTA 8.9%; SNHL 37.5%; mixed 37.5%; mild 37.5%; mod 25%; Sev 25%; Prof 12.5%	21/97 (21.6%)	DM + HTN; glycaemic control
Akbar et al. 2019 [27]	India	Cross‐sectional, comparative	ENT Dept., Govt med College	July 2016–June 2017 (1 year)	*N* = 126 (DM 63; control 63); 30–60 years; %F NR	Type 2	PTA	126	Normal 22.2%; minimal 4.8%; mild 7.9%; mod 17.5%; Mod‐Sev 38.1%; Sev 9.5%	Normal 63.5%; minimal 25.4%; mild 11.1%	49/63 (77.8%)	NR
Al‐Sofiani et al. 2020 [28]	Saudi Arabia	Cross‐sectional	Endocrine clinic and audiology labs	NR	*N* = 41 (DM 30; control 11); DM 43.8 years, 50% F; control 53 years	Type 1	PTA	41	HF‐ HL associated with ↑age, longer DM duration, neuropathy	NR	19/30 (63.3%)	Age, duration, peripheral neuropathy
Asghar et al. 2024 [29]	Pakistan	Cross‐sectional	Diabetes clinic	May–September 2021 (5 months)	*N* = 396; mean 48.6 years; 77% F	Type 2	PTA (air & bone)	396	4fPTA: R 34.93 dB, L 34.57 dB; normal 25.3%; mild 55.8%; mod 18.7%	NA	296/396 (74.7%)	HL severity ↑ with MNSI (neuropathy) (OR 0.23, 0.11–0.48) *
Bamanie and Al‐Noury 2011 [41]	Saudi Arabia	Case–control	Univ. hospital ENT	January 2005–December 2009 (4 years)	*N* = 196 (DM 109; control 87); DM 47.9 years, 52% F; control 45.7 years, 47% F	Type 2	PTA; flat audiograms	196	Worst ear HL 69.7%; best ear HL 52.3%	Worst ear HL 39.1%; best ear HL 25.3%	Worst ear 76/109 (69.7%)	Age; DM complications; insulin therapy; duration NS
Chee et al. 2022 [50]	Singapore	Cross‐sectional (retrospective)	Community hearing clinic	November 2017–November 2018 (1 year)	*N* = 1787; mean 71.3 years; 52.5% F; 17.6% with DM	Type 2	Audiological assessment, PTA	1787	Normal 28%; mild 42.3%; Mod+ 29.7% (overall)	NR	Mod + HL adj OR 1.27 (1.05–1.54)	Age, male sex, HTN, HLD, noise exposure
Cheng et al. 2009 [42]	United States	Cross‐sectional (NHANES)	National survey clinics	NHANES I 1971–1973; 1999–2004	*N* = 3192 & 4486; ∼51% F; mean ∼44 years	Type 1 & 2 (self‐report)	PTA	3192; 4486	Adj Prev 28.5% (NHANES I); 34.4% (1999–2004)	Adj Prev 28.5% (NHANES I); 34.4% (1999–2004)	Adj PR 1.17 → 1.53 across surveys	Age, male, white race, lower SES
Dhasmana et al. 2021 [30]	India	Cross‐sectional	Hospital (ENT)	12 months (NR)	*N* = 200 T2D; mean 47 years; 45.5% F	Type 2	PTA, tuning fork, ENT exam	200	Mean HL 34.1 dB; SNHL 79%; mixed 12%; conductive 9%; mild 66.3%; mod 30.9%; Sev 2.7%	NA	110/200 (55%)	↑FBS, ↑HbA1c; duration NS; complications NS
Esubalew et al. 2024 [31]	Ethiopia	Multi‐centre cross‐sectional	Hospitals	May–June 2022 (6 weeks)	*N* = 846; mean 51.6 years; 41.2% F	Type 2	PTA	846	Bilat 75.5%; unilateral HL 24.5%; mild 55.6%; mod 26.1%; Mod–Sev 14%; Sev 4.3%	NA	SNHL 427/846 (50.5%; 95% CI 45.7–55.3)	Age, duration ≥ 10 years, HTN, HLD; exercise protective
Gong et al. 2018 [43]	China	Cross‐sectional (community survey)	Community; trained GPs; quiet rooms < 40 dBA	August 2014–September 2015 (1 year)	*N* = 6984 ≥ 60 years; 52.2% F (DM 388; control 6596)	Type 1 & 2 (self‐report/meds)	PTA: WHO protocol	6984	HL 71.9%; mild 53.8%; mod 32.3%; Sev 10.0%; Prof 3.9%	HL 58.1% (3831/6596)	279/388 (71.9%); OR 1.85 (1.47–2.32)	Age, male, ear disease, DM, HTN, AS, noise, autotoxins
Helzner et al. 2005 [44]	United States	Longitudinal cohort	Health ABC clinics	2001–2002 (year‐5 visit)	*N* = 2052; mean 77.5 years; 52.7% F (DM 371)	Type 1 & 2 (self‐report)	PTA, otoscopy	2052	HL 66.8% (PTA 0.5–2 k > 25 dB); HF‐only HL 12.4%	HL 58.4%; HF‐only 21.7%	248/371 (66.8%)	DM aOR 1.42 (1.10–1.83); plus, CVD, smoke, noise, ear surgery
Hlayisi et al. 2019 [32]	South Africa	Cross‐sectional (matched)	Hospital	NR	*N* = 192 (cases 110; control 82); 18–55 years; ∼51% F	Type 1 & 2	PTA + DPOAE	192	SNHL 74%; slight 35%; mild 26%; mod 32%; Sev 7%	SNHL 67%; slight 44%; mild 25%; mod 22%; Sev 9%	55% (cases 53/96?)	Duration OR 1.12; age OR 2.90; male ↑; control NS
Jordan et al. 2022 [33]	United States	Cross‐sectional (NHANES 1999–2002)	National survey	1999–2002 (4 years)	*N* = 3409; 20–69 years; 51.1%	NR	PTA, otoscopy, tympanometry	3409	Slight 31.3%; mild 27.4%; mod 11.2% (overall)	NA	Adj OR 1.67 (1.21–2.31)	Age, male, HTN, arthritis
Kabeya et al. 2013 [34]	Japan	Cross‐sectional	Health check‐up	May 2008–December 2010	*N* = 2753; DM 483 (64.1 yrs, 15.9% F); non‐DM 2270 (56.6 years, 36.3% F)	NR	PTA	2753	High‐frequency HI adj OR 1.33 (1.02–1.74)	NR	HF HI: DM 26.7%; non‐DM 12.1%	Age (≥ 70 OR 32.6), male (OR 2.4), smoking
Lee et al. 2023 [35]	South Korea	Retrospective case–control	Catholic medical centre	2006–2021 (15 years)	*N* = 5287; normal 1129; PreDM 2119; DM 2039; mean age 64–70; ∼51%–58% F	NR (based on HbA1c groups)	PTA; speech (SDS)	5287	Mean PTA (note: values inverted in text); total HL *n* = 2606	See groups	Normal 25.8%; PreDM 40.6% (aOR 1.12 ns); DM 33.6% (aOR 1.47)	Higher HbA1c worsened thresholds; HF 8 kHz aOR 1.35
Li et al. 2020 [36]	China	Case–control	Hospital	March–August 2017 (6 months)	*N* = 125 (DM 65; control 60); ∼45.5 years; ∼44% F	Type 2	PTA, high‐frequency audiometry, DPOAE	125	HF threshold 37.7 dB; DPOAEs lower ≥ 1 kHz	HF threshold 29.3 dB	41/65 (63.1%)	Age; duration ≥ 10 years ↑ thresholds; HbA1c NS
Mishra et al. 2024 [45]	India	Cross‐sectional	Tertiary hospital OPD	NR	*N* = 152 (< 60 years); mean 49 years; 44.1% F	Type 2	Otoscopy, tymp, PTA, OAE	152	SNHL 70.4%; mild 62.6%; mod 33.6%; Sev‐Prof 3.7%; HF pattern 67%	NA	107/152 (70.4%)	Rural, FHx DM, duration, HbA1c, complications
Mitchell et al. 2009 [46]	Australia	Longitudinal cohort	Community survey	1997–2004 (7 years)	*N* = 1858 (DM 210; non‐DM 1648); ∼70 years; ∼54%–58% F	Type 2	PTA; HHIE scale	1858	HL 50%; mod 32.4%; Sev 15.7%	HL 38.2%; mod 25.5%; Sev 10.7%	105/210 (50%); OR 1.55 (1.11–2.17)	Duration ≥ 10 years OR 2.08; incident HL similar; age, male, noise, low educated levels
Mozaffari et al. 2010 [47]	Iran	Cross‐sectional (matched controls)	Clinic/ENT	NR	*N* = 160 (DM 80; control 80); mean 45 years; 63.8% F	Type 1 and 2	PTA (air and bone)	160	SNHL 45%; laterality: Uni 33%; bi 67%; mild 39%; mod 47%; Mod‐Sev 8%; Sev 3%; Prof 3%	SNHL 20%; mild 56%; mod 38%; Mod‐Sev 6%	36/80 (45%); OR 3.5 (1.6–6.6)	Within DM: longer duration; lower age at onset; HbA1c NS; DM complications NS
Naik and Tilloo 2018 [53]	India	Cross‐sectional	Rural tertiary hospital	NR	*N* = 100; 30–60 years; 38% F	Type 2	PTA, OAE, vestibular tests	100	SNHL 69%; grades: Slightly 46%; mild 7%; mod 28%; mod–Sev 14%; Sev 5%	NA	69/100 (69%)	Worse with poorer HbA1c; age ↑; females higher
Nkosi et al. 2024 [48]	South Africa	Cross‐sectional	District hospital OPD	NR	*N* = 35 (70 ears); mean 41 years; 57% F; DM < 10 years	Type 2	Otoscopy, tymp, PTA (air/bone), EHF, SRT, DPOAE, ABR	35	22/70 ears (31.4%); degree mix across ears	NA	31.4% (per ear)	HTN assoc.; HbA1c NS; duration NS; sex NS
Ozkurt et al. 2016 [54]	Turkey	Case–control	Hospital	January–June 2014 (6 months)	*N* = 80 (DM 40; control 40); 40–50 years; ∼51% F	Type 2	PTA + high‐frequency audiometry	80	Higher thresholds all frequency; R 11.2 k: 33.8 dB; 16 k: 38.5 dB	R 11.2 k: 9.5 dB; 16 k: 17.6 dB	NR	Thresholds ↑ with longer DM duration
Pathak et al. 2017 [37]	India	Prospective	Hospital	NR	*N* = 100; 31–55 years; 38% F	Type 2	PTA	100	SNHL: Slight 47%; mild 37%; mod 17%	NA	60/100 (60%)	Longer duration ↑ severity; HbA1c > 8% assoc.
Ren et al. 2017 [38]	China	Case–control	Hospital	September 2013–March 2014 (8 months)	*N* = 260 (DM 160; control 100); ∼51 years; 31%–39% F	Type 2	PTA	260	Higher thresholds at 2–8 kHz; HF impairment 48.8%; low/midHF 18.8%	NR	108/160 (67.5%)	↑MNSI, ↑VPT, ↑SWM; age ↑
Sakuta et al. 2007 [49]	Japan	Cross‐sectional	SDF retirement health check	NR	*N* = 699; mean 52.9 years; 0% F; DM 103	Type 2	PTA (air and bone)	699	Mean HL 30.7 dB; HF HL 43.7 dB; HL > 25 dB 60.2%; HF HL 76.7%; LF HL 34.0%	HL > 25 dB 45.2% (NGT)	60.2% (DM); NGT 45.2%	DM aOR 1.87; hyperTG; heavy drinking
Shaikh et al. 2020 [39]	Pakistan	Case–control	University hospital	February–July 2017 (6 months)	*N* = 196 (DM 98; control 98); 20–40 years; %F NR	Type 2	PTA	196	Normal 65; slight 32; mod 1	Normal 97; slight 1	34% slight–mod	NR
Srinivas et al. 2016 [52]	India	Cross‐sectional	Hospital ENT and Medicine	November 2014–October 2015 (1 year)	*N* = 50 T2D; 31–65 years; 71.4% F	Type 2	Tuning fork, PTA, impedance	50	SNHL 66%; mild 54%; mod 12%	NA	33/50 (66%)	Age, duration, HbA1c, FBS
Uchida et al. 2010 [51]	Japan	Cross‐sectional (community)	National centre Geriatrics and Gerontology	May 2004–July 2006 (2 years)	*N* = 2306; age groups 40–64 & 65–86; ∼50% F	Type 1 & 2	PTA	2306	DM+: HF HL (> 25 dB BE): 44.8% (40–64) & 79.8% (65–86)	HF HL: 16.8% (40–64) & 75.1% (65–86)	See age strata; CI NR	DM impaired most freqs; DM × Age at 4 & 8 kHz
Vesperini et al. 2011 [40]	Italy	Case–control	Hospital	NR	*N* = 60 (DM 40; control 20); age ≥ 20	Type 2	PTA, OAE, BAEP, impedance	60	PTA (500–8000 Hz) mean 14.23 dB	PTA mean 7.45 dB	83% (definition per study)	HF (4–8 kHz) most affected; reduced OAE

*Note:* Prevalence values follow each study’s definitions; some studies report per‐ear versus per‐person metrics. Severity thresholds typically follow WHO/ASHA (mild 26–40 dB HL; moderate 41–60 dB HL; severe 61–80 dB HL; profound ≥ 81 dB HL).

Abbreviations: ABR = auditory brainstem response, aOR = adjusted odds ratio, CI = confidence interval, DPOAE = distortion product otoacoustic emissions, HF = high frequency, HL = hearing loss, HLD = hyperlipidaemia, HTN = hypertension, LF = low frequency, NR = not reported, OR = odds ratio, PreDM = prediabetes, PTA = pure‐tone audiometry, SDS = speech discrimination score, SNHL = sensorineural hearing loss.

### 3.3 Pooled Prevalence of Moderate‐to‐Severe Hearing Loss in Diabetes

**FIGURE 2:**
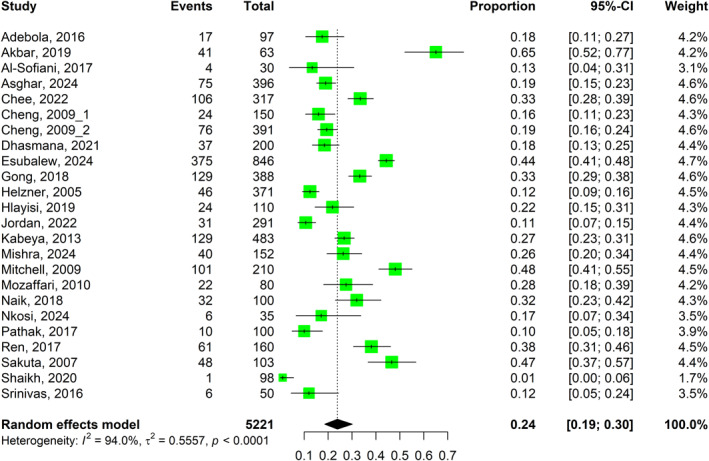
Forest plot of moderate‐to‐severe (≥ 40 dB HL) hearing loss prevalence among individuals with diabetes.

**FIGURE 3:**
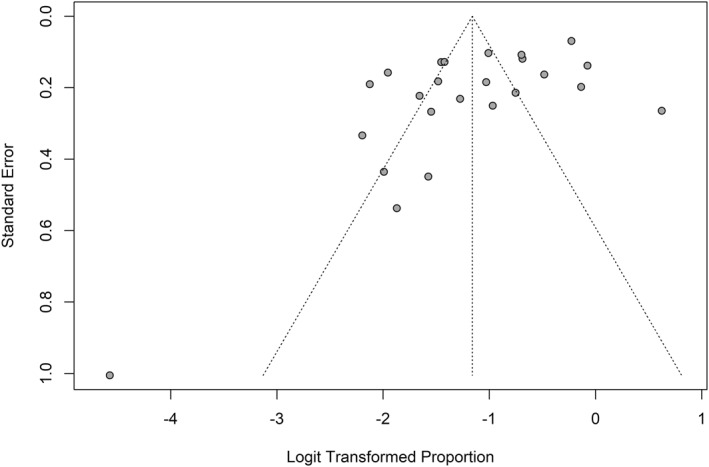
Funnel plot assessing publication bias for prevalence estimates.

Twenty‐three studies provided data suitable for pooling (Supporting Information S5). The pooled prevalence of moderate‐to‐severe (≥ 40 dB HL) hearing loss among individuals with diabetes was 24% (95% CI: 19%–30%), equivalent to approximately one in four adults (Figure 2). Heterogeneity was substantial (*I* ^2^ = 94%), reflecting variation in population characteristics, measurement methods, and sample sizes. Funnel plot inspection (Figure 3) revealed moderate asymmetry, and Egger's test confirmed small‐study effects (*t* = −2.54, *p* = 0.019). Leave‐one‐out sensitivity analyses demonstrated that no single study substantially altered the overall conclusions. Removing high‐effect or small‐sample studies slightly attenuated pooled estimates but did not change the direction or statistical significance of associations. These results confirm the robustness of the observed relationship between diabetes and clinically significant hearing loss despite high heterogeneity. Prediabetes (vs. normoglycaemia) showed no significant increase in the odds of ≥ 40 dB hearing loss in the single study reporting this comparison (adjusted OR = 1.12; 95% CI includes 1); evidence remains insufficient for pooling.

### 3.4 Comparative Risk Moderate‐to‐Severe Hearing Loss: Diabetes Versus Non‐Diabetic Controls

**FIGURE 4:**
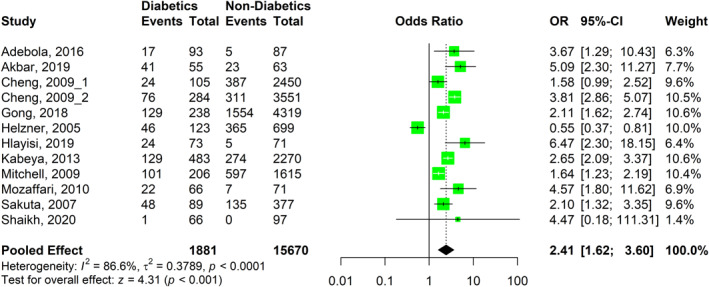
Forest plot of pooled odds ratios for moderate‐to‐severe hearing loss: Diabetes versus non‐diabetic controls.

Eleven studies (*n* = 17,051 participants; 1881 with diabetes, 15,670 non‐diabetic controls) provided comparative data enabling quantification of the association between diabetes and moderate‐to‐severe hearing loss [26, 27, 32, 34, 39, 42, 43, 44, 46, 47, 49]. Notably, one study [42] reported two independent datasets (Cheng, 2009_1 and Cheng, 2009_2), resulting in 12 distinct comparisons from 11 publications. The pooled odds ratio demonstrated that individuals with diabetes had significantly elevated odds of clinically significant hearing impairment compared with non‐diabetic controls (OR = 2.41, 95% CI: 1.62–3.60, *p* < 0.001), representing more than a two‐fold increase in risk (Figure 4). This association remained statistically significant and robust across multiple analytical approaches.

**FIGURE 5:**
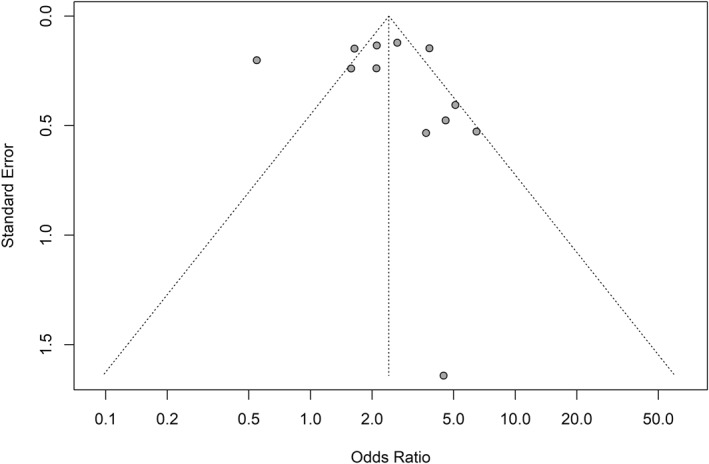
Funnel plot assessing publication bias for odds ratio estimates.

Individual study estimates ranged from OR = 0.55 [44] to OR = 6.47 [32], with most comparisons (10 of 12) reporting odds ratios between 1.5 and 5.0. Substantial heterogeneity was observed (*I* ^2^ = 86.6%, *τ* ^2^ = 0.38, *p* < 0.0001). Sensitivity analyses excluding outliers yielded similar results (OR = 2.2), confirming robustness. Egger’s test indicated small‐study effects (*p* = 0.019), and funnel plot asymmetry suggested potential publication bias (Figure 5). However, the association remained consistently positive across nearly all studies (except [44]) and statistically significant despite this limitation (*z* = 4.31, *p* < 0.001).

### 3.5 Effect Modification by Age Group

**FIGURE 6:**
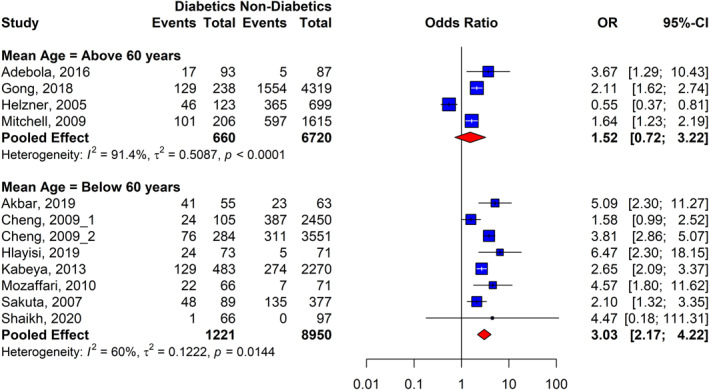
Forest plot of diabetes‐associated hearing loss stratified by age group (< 60 vs. ≥ 60 years).

Age‐stratified analyses examined whether the diabetes‐hearing loss association varied across age categories, revealing differential patterns between younger and older adults. Among individuals below 60 years of age, eight studies (*n* = 10,171 participants) demonstrated a strong and statistically significant association, with a pooled odds ratio of 3.03 (95% CI: 2.17–4.22, *p* < 0.001; Figure 6). This subgroup exhibited moderate heterogeneity (*I* ^2^ = 60%, *τ* ^2^ = 0.12, *p* = 0.01), suggesting some variation in effect sizes across studies despite consistent directionality. Individual study estimates within this younger cohort ranged from OR = 1.58 [42] to OR = 6.47 [32], with most studies reporting odds ratios between 2.0 and 5.0. In contrast, among adults aged 60 years and above, four studies (*n* = 7380 participants) yielded a pooled odds ratio of 1.52 (95% CI: 0.72–3.22), which was not statistically significant (Figure 6). This older age subgroup displayed substantial heterogeneity (*I* ^2^ = 91.4%, *τ* ^2^ = 0.51, *p* < 0.001), reflecting considerable inconsistency across study findings. Individual study estimates ranged widely from OR = 0.55 to OR = 3.67 [26, 44].

### 3.6 Effect Modification by Country Income Level

**FIGURE 7:**
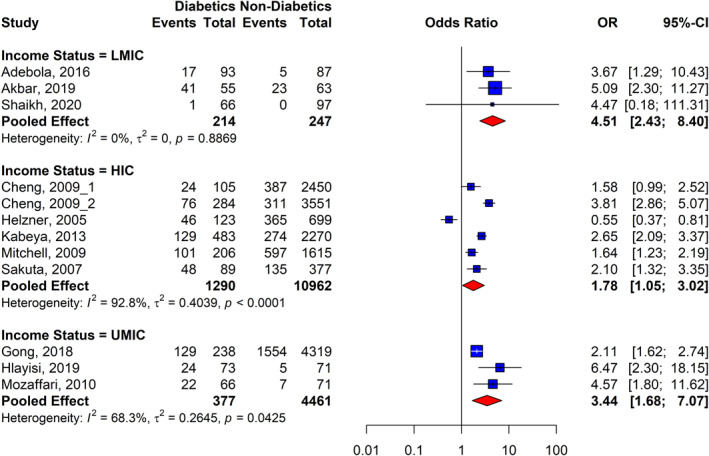
Forest plot of diabetes‐associated hearing loss stratified by national income level (LMICs, UMICs, HICs).

When stratified by World Bank income classification, the association between diabetes and moderate‐to‐severe hearing loss demonstrated variation across economic settings. All three income categories showed statistically significant associations when compared to non‐diabetic controls (Figure 7). Low‐ and middle‐income countries (LMICs) exhibited the highest pooled odds ratio (OR = 4.51, 95% CI: 2.43–8.40; 3), based on data from studies conducted in Nigeria, Pakistan, and India [26, 27, 39]. Upper‐middle‐income countries (UMICs) showed intermediate risk (OR = 3.44, 95% CI: 1.68–7.07). High‐income countries (HICs) demonstrated the lowest, though still statistically significant, association (OR = 1.78, 95% CI: 1.05–3.02), including studies from the United States, Australia, and Japan [34, 42, 44, 46, 49].

### 3.7 Duration of Diabetes

**FIGURE 8:**
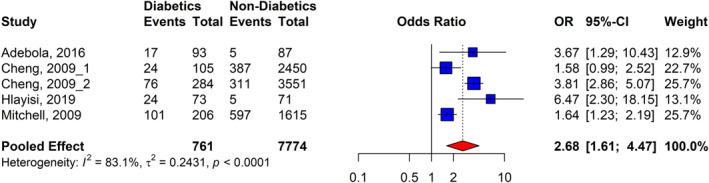
Forest plot of diabetes‐associated hearing loss stratified by diabetes duration (< 10 years).

Five studies reported outcomes stratified by diabetes duration. Individuals with a disease duration of less than 10 years had significantly increased odds of moderate‐to‐severe hearing loss compared with non‐diabetics, with a pooled odds ratio (OR) of 2.68 (95% CI: 1.61–4.47; *I* ^2^ = 83.1%, *p* < 0.0001; Figure 8). The individual studies ORs range from 1.58 to 6.47. One study that examined diabetes duration ≥ 10 years reported an even stronger association (OR = 4.57, 95% CI: 1.80–11.62), although the data were insufficient for pooled analysis [47].

## 4 Discussion

This systematic review and meta‐analysis provide the first comprehensive synthesis specifically examining the pooled prevalence and comparative risk of clinically significant moderate‐to‐severe hearing loss (≥ 40 dB HL) in adults with diabetes. Our findings reveal that nearly one in four adults with diabetes experiences this level of hearing impairment, and that diabetes more than doubles the odds of functionally disabling hearing loss (pooled OR = 2.41, 95% CI: 1.62–3.60), with a disproportionate burden observed among younger adults and populations in LMICs.

The observed 24% pooled prevalence of moderate‐to‐severe hearing loss (≥ 40 dB HL) in our study represents a clinical burden comparable to other well‐established diabetic microvascular complications such as retinopathy and nephropathy [57]. This distinction from prior meta‐analyses, which often encompassed hearing loss of any severity (e.g., a reported 53% measurable hearing loss in type 2 diabetes versus 25% in controls) [13]. Our exclusive focus on the ≥ 40 dB HL threshold is critical because it precisely delineates impairment that critically disrupts daily communication, social engagement, and occupational productivity. Unlike mild loss, this level compromises understanding speech in noisy settings, leading to functional disability, increased social isolation and depression [58], and is associated with accelerated cognitive decline [59]. Such significant impairment necessitates audiological intervention.

Age‐stratified analyses revealed contrasting patterns: adults below 60 years demonstrated a significant association with moderate‐to‐severe hearing loss (OR = 3.03, 95% CI: 2.17–4.22), whereas those aged ≥ 60 years showed no significant association (OR = 1.52, 95% CI: 0.72–3.22). These findings require cautious interpretation due to potential confounding by presbycusis. Inconsistent control for age‐related hearing loss across studies, highly prevalent beyond age 60, may introduce confounding effects, particularly in older cohorts where age‐related decline could obscure diabetes‐specific effects [16]. The non‐significant finding in older adults may therefore reflect age‐related hearing loss overshadowing diabetes effects, inadequate age‐matching between comparison groups, or limited sample size. Conversely, the robust association in younger adults, where physiological hearing decline is minimal, suggests that diabetes may operate as a more prominent independent risk factor in this demographic [60]. These patterns suggest that younger adults with diabetes warrant consideration for targeted audiometric screening. Early detection enables timely intervention before substantial impairment manifests, potentially yielding long‐term benefits given extended life expectancy and greater occupational engagement [17, 61].

The observed socioeconomic gradient demonstrates that risk was elevated across all income settings, with the highest odds in low‐ and middle‐income countries (OR = 4.51, 95% CI 2.43–8.40) and upper‐middle‐income countries (OR = 3.44, 95% CI 1.68–7.07), compared to high‐income countries (OR = 1.78, 95% CI 1.05–3.02). This gradient could reflect multiple converging factors. First, suboptimal glycaemic control resulting from limited access to diabetes medications and monitoring equipment may drive increased microvascular complications [62]. Second, a higher comorbidity burden and more advanced microvascular damage at diagnosis may synergistically exacerbate auditory impairment [16]. Third, delayed detection arising from inadequate screening programmes and financial barriers may prevent timely intervention [10]. Additionally, greater unregulated occupational noise exposure in resource‐limited settings may compound diabetes‐related effects [18]. Alternative explanations warrant consideration, including variations in audiometric methodology, differences in ototoxic medication exposure, or unmeasured confounding factors. This pattern highlights global health equity concerns. Individuals with diabetes in LMICs have demonstrated an elevated risk yet often lack access to audiological services and hearing aids [29]. However, these observational findings require confirmation through standardised methodology, as alternative explanations cannot be excluded. If income‐level differences are substantiated, affordable hearing screening could be integrated into LMIC diabetes care through primary care or community health worker models [63].

Despite limited data, our duration analysis provides preliminary evidence regarding the relationship between diabetes duration and moderate‐to‐severe hearing loss. A single study reported elevated odds among those with diabetes duration ≥ 10 years (OR = 4.57), while pooled analysis of multiple studies with duration < 10 years yielded a lower estimate (OR = 2.68). With only one study examining ≥ 10 years, formal statistical comparison between duration categories was not feasible, and the apparent gradient may reflect study‐specific factors rather than a true dose‐response relationship [64]. Our finding aligns with and strengthens observations from other studies that generally reported an increased likelihood of hearing loss with longer duration of diabetes, though often with varying definitions of hearing loss severity [11, 12]. Earlier evidence also linked prolonged hyperglycaemia to cochlear microangiopathy [65].These findings suggest a case for incorporating hearing screening at diabetes diagnosis, rather than waiting for advanced disease [66]. Longitudinal studies with standardised duration categorisation are essential to clarify whether diabetes duration independently predicts hearing loss severity after accounting for glycaemic control and other metabolic factors.

Key limitations of our analysis include the following. First, substantial heterogeneity (*I* ^2^ = 86.6% for OR analysis) reflects variation in study populations, diabetes type and adjustment for confounders such as noise exposure and ototoxic medications. The decision to pool these studies, despite substantial statistical heterogeneity (*I* ^2^ = 86.6%), was based on the consistent direction of the observed effect across nearly all included studies, which suggests a robust underlying association between diabetes and hearing loss. In line with Cochrane guidelines, which caution against pooling primarily when there is inconsistency in the direction of effect rather than variation in magnitude, we determined that a meta‐analysis was both appropriate and clinically informative [25]. The observed heterogeneity likely reflects true clinical and epidemiological differences between the diverse populations studied, such as variations in age, diabetes duration, and healthcare settings—rather than methodological flaws. By employing a random‐effects model, we have statistically accounted for this between‐study variance, and the resulting pooled estimate should be interpreted as the mean of a distribution of risks across settings rather than a single precise value for any specific population. This approach is consistent with precedent in high‐impact meta‐analyses, where clinical consistency in the direction of effect justifies pooling despite high *I* ^2^ values [11, 13].

A portion of the observed heterogeneity is also attributable to methodological variance in audiometric definitions. While our analysis prioritised the WHO standard of using the better ear average over speech frequencies (0.5–4 kHz) [56], some included studies deviated from this approach [36, 41]. For instance, Bamanie (2011) utilised ‘worse ear’ criteria, which can inflate prevalence estimates compared to the ‘better ear’ method. These variations contribute to the statistical heterogeneity but also reflect the real‐world diversity of clinical practice and research methodology. Importantly, previous large‐scale meta‐analyses have conducted subgroup analyses on these very factors, speech frequencies versus the full audiometric range, and unilateral versus bilateral criteria, and found that they did not significantly modify the association between diabetes and hearing loss [11]. Although our dataset was insufficient to formally test each of these variations, the consistency of our overall findings with prior research suggests that the core association is robust to these definitional differences.

Secondly, publication bias, evidenced by funnel plot asymmetry and Egger’s test (*p* = 0.019), suggests that small studies with null findings may be underrepresented; however, sensitivity analyses confirmed the robustness of our core findings. Thirdly, the predominantly cross‐sectional design of the included studies precludes definitive causal inference, though the biological plausibility of diabetes impacting cochlear microvasculature and neural pathway integrity, coupled with consistency across studies, strongly supports a causal relationship. Finally, data on prediabetes remain limited to a single study, precluding definitive conclusions about the risk of hearing loss in this population. Future research employing standardised audiometric criteria (e.g., using pure‐tone audiometry across multiple frequencies rather than self‐report or screening tools) and longitudinal designs will be crucial to refine risk magnitude and identify the most affected subgroups, ultimately guiding more precise screening and intervention strategies.

Taken together, these findings underscore the need to recognise hearing loss as a clinically significant and prevalent complication of diabetes that warrants greater attention in both clinical practice and public health policy. The elevated risk observed in younger adults challenges the assumption that diabetes‐related hearing loss is merely an acceleration of age‐related decline, while the striking socioeconomic gradient highlights an urgent global health equity concern. Integrating hearing assessments into routine diabetes management—particularly in low‐ and middle‐income countries where the burden is greatest and access to audiological services is most limited—represents a tangible opportunity to reduce disability burden and improve quality of life for millions of individuals living with diabetes worldwide.

## 5 Conclusion

In conclusion, this systematic review and meta‐analysis demonstrates that moderate‐to‐severe hearing loss (≥ 40 dB HL) affects nearly one in four adults with diabetes, representing a significant, yet under‐recognised, complication. Crucially, this sensory impairment is not merely an audiometric finding but a functionally disabling condition that impairs communication, increases social isolation, and accelerates cognitive decline. The more than two‐fold increased risk identified in this review supports the integration of routine audiometric screening into standard diabetes care, with particular urgency for younger adults and those in low‐ and middle‐income countries, where the burden appears greatest. Early detection through systematic screening could facilitate timely intervention, offering a crucial opportunity to prevent progression and significantly improve communicative function and overall quality of life for individuals living with diabetes.

## Author Contributions

M.N., S.K., A.R., B.N.A., and P.D. contributed to the conceptualisation and study design. M.N., S.K., and A.R. conducted the systematic literature search, classified the retrieved information, and performed the initial synthesis of the findings. M.N. and A.R. undertook the formal analysis, data verification, and visualisation, while B.N.A. and P.D. provided intellectual input and critical oversight of the analytical process. M.N. prepared the original draft of the manuscript. S.K., A.R., B.N.A., and P.D. critically reviewed and edited the manuscript. All authors have read and approved the final version of the manuscript.

## Funding

The authors have nothing to report.

## Conflicts of Interest

The authors declare no conflicts of interest.

## Supporting information


Supporting Information S1

Supporting Information S2

Supporting Information S3

Supporting Information S4

Supporting Information S5

## Data Availability

Data sharing not applicable to this article as no datasets were generated or analysed during the current study.
