# Supplementary material for: Hearing Loss in Adults With Diabetes and Prediabetes: A Systematic Review and Meta‐Analysis
Source: Diabetes Metab Res Rev. 2026 Jun 23;42(5):e70195. doi: 10.1002/dmrr.70195 (PMC13288450; doi:10.1002/dmrr.70195)
Supplement: Supplementary file 3 — Supporting Information S3 [file DMRR-42-e70195-s003.docx]

Supplementary 3

**Table: Search strategy for studies investigating hearing screening and diabetes across five electronic databases (search conducted 31^ST^ August 2025)**

| **Database** | **Date Searched** | **Search Strategy (as executed)** | **Results** |
| --- | --- | --- | --- |
| **PubMed (MEDLINE)** | August 2025 | (“hearing screening” OR “screening, auditory” OR “auditory screening” OR “hearing screen*” OR “auditory screen*” OR “audiological screen*” OR “audiometric screening” OR “audiometric assessment” OR (hear* AND “health check-up”)) **AND** (diabetes OR prediabetes OR “chemical diabetes” OR “latent diabetes” OR “prediabetic state” OR “impaired glucose tolerance” OR related synonyms). Search fields: [Title/Abstract], [All Fields]. Controlled vocabulary: MeSH terms “Diabetes Mellitus,” “Prediabetic State.” | 1178 |
| **Embase (Elsevier)** | August 2025 | (‘auditory screening’/exp OR ‘hearing screening’ OR ‘screening, auditory’ OR (hear* NEAR/4 screen*) OR (auditory NEAR/4 screen*) OR (audiological NEAR/4 screen*) OR (audiometric NEAR/4 assessment) OR (audiometric NEAR/4 screen) OR (hear* AND ‘health check-up’)) **AND** (‘diabetes mellitus’/exp OR ‘impaired glucose tolerance’/exp OR diabet* OR prediabet* OR “chemical diabetes” OR “latent diabetes” OR “prediabetic state” OR “impaired glucose tolerance” OR related synonyms). Controlled vocabulary: Emtree terms ‘hearing’/exp, ‘screening’/exp, ‘diabetes mellitus’/exp, ‘impaired glucose tolerance’/exp. | 920 |
| **Cochrane Library (CENTRAL)** | August 2025 | #1 (‘hearing screening’ OR ‘screening, auditory’ OR ‘auditory screening’):ti,ab,kw #2 ((hear* NEAR/4 screen*) OR (auditory NEAR/4 screen*) OR (audiological NEAR/4 screen*) OR (audiometric NEAR/4 assessment) OR (audiometric NEAR/4 screen)):ti,ab,kw #3 #1 OR #2 #4 MeSH descriptor: [Diabetes Mellitus] explode all trees #5 MeSH descriptor: [Diabetes Insipidus] explode all trees #6 MeSH descriptor: [Prediabetic State] explode all trees #7 (diabet* OR prediabet* OR related synonyms):ti,ab,kw #8 OR #4–#7 #9 #3 AND #8 | 129 |
| **CINAHL (EBSCOhost)** | August 2025 | S1 (MM “Hearing Screening”) S2 “hearing screening” OR “screening, auditory” OR (hear* N4 screen*) OR (auditory N4 screen*) OR (audiological N4 screen*) OR (audiometric N4 assessment) OR (audiometric N4 screen) S3 hear* AND “health check-up” S4 S1 OR S2 OR S3 S5 (MM “Diabetes Mellitus+”) S6 (MM “Diabetes Insipidus+”) S7 (MM “Prediabetic State”) S8 diabet* OR prediabet* OR related synonyms S9 S5 OR S6 OR S7 OR S8 S10 S4 AND S9 Controlled vocabulary: CINAHL Headings (MM terms). | 134 |
| **Scopus (Elsevier)** | August 2025 | ((TITLE-ABS-KEY (“hearing screening” OR “screening, auditory” OR “auditory screening” OR (hear* W/4 screen*) OR (auditory W/4 screen*) OR (audiological W/4 screen*) OR (audiometric W/4 assessment) OR (audiometric W/4 screen))) OR (TITLE-ABS-KEY (hear* AND “health check-up”))) **AND** ((TITLE-ABS-KEY (diabet* OR prediabet*)) OR (TITLE-ABS-KEY (“chemical diabetes” OR “latent diabetes” OR “impaired glucose tolerance” OR related synonyms))). | 531 |
| **Web of Science (Core Collection)** | August 2025 | TS=(‘hearing screening’ OR ‘screening, auditory’ OR ‘auditory screening’) TS=((hear* NEAR/4 screen*) OR (auditory NEAR/4 screen*) OR (audiological NEAR/4 screen*) OR (audiometric NEAR/4 assessment) OR (audiometric NEAR/4 screen)) TS=(hear* AND “health check-up”) #4 = #1 OR #2 OR #3 TS=(diabet* OR prediabet* OR “chemical diabetes” OR “latent diabetes” OR “prediabetic state” OR “impaired glucose tolerance” OR related synonyms) #7 = #5 OR #6 #8 = #7 AND #4 | 598 |

**Notes:**

1. **Controlled vocabulary terms** were applied where available:
   - *PubMed*: MeSH (Medical Subject Headings).
   - *Embase*: Emtree terms.
   - *CINAHL*: CINAHL Subject Headings (MM terms).
   - *Cochrane*: MeSH descriptors.
2. **Boolean operators** (AND, OR) were used to combine hearing-related and diabetes-related concepts.
3. **Proximity operators**:
   - NEAR/n (Embase, Cochrane, Web of Science, CINAHL) and W/n (Scopus) restricted word proximity within *n* terms.
4. **Truncation symbol** * was used to capture word variants (e.g., *diabet* retrieves diabetes, diabetic, diabetics).
5. Searches were limited to **title, abstract, and keyword fields** where applicable.
6. No date, language, or publication-type restrictions were applied at this stage.
