# Supplementary material for: Hearing Loss in Adults With Diabetes and Prediabetes: A Systematic Review and Meta‐Analysis
Source: Diabetes Metab Res Rev. 2026 Jun 23;42(5):e70195. doi: 10.1002/dmrr.70195 (PMC13288450; doi:10.1002/dmrr.70195)
Supplement: Supplementary file 4 — Supporting Information S4 [file DMRR-42-e70195-s002.docx]

Supplementary 4

**Quality assessment of systematic reviews/meta-analyses using the Newcastle-Ottawa Scale (NOS).**

References:

- Ga, W. *The Newcastle-Ottawa Scale (NOS) for assessing the quality of nonrandomised studies in meta-analyses*. in *3rd Symposium on Systematic Reviews: Beyond the Basics, Oxford, UK, 3-5 July 2000*. 2000.
- Forte, A.J., et al., *The Impact of Optimism on Cancer-Related and Postsurgical Cancer Pain: A Systematic Review.* Journal of Pain and Symptom Management, 2022. **63**(2): p. e203-e211.
