# Supplementary material for: Hearing Loss in Adults With Diabetes and Prediabetes: A Systematic Review and Meta‐Analysis
Source: Diabetes Metab Res Rev. 2026 Jun 23;42(5):e70195. doi: 10.1002/dmrr.70195 (PMC13288450; doi:10.1002/dmrr.70195)
Supplement: Supplementary file 5 — Supporting Information S5 [file DMRR-42-e70195-s004.docx]

Supplementary 5

**Table: Summary of Extracted Data for Meta-Analysis: Diabetes and Clinically Significant Hearing Impairment**

| **Study** | **Total N** | **Overall HL** | **Total Cases** | **Cases with HL** | **Cases without HL** | **Moderate-Severe HL cases** | **Total Controls** | **Controls with HL** | **Controls without HL** | **Moderate-Severe controls** | **Age (mean/range)** | **Age category** | **Country** | **Income status** | **Diabetes duration years (mean)** | **Duration category** |
| --- | --- | --- | --- | --- | --- | --- | --- | --- | --- | --- | --- | --- | --- | --- | --- | --- |
| Adebola, 2016 | 187 | 29 | 97 | 21 | 76 | 17 | 90 | 8 | 82 | 5 | 58 | Above 60 | Nigeria | LMIC | 7.6 (6.4) | Below 10 |
| Akbar, 2019 | 126 | 72 | 63 | 49 | 14 | 41 | 63 | 23 | 40 | 23 | 30-60 | Below 60 | India | LMIC | NA | NA |
| Al-Sofiani, 2017 | 41 | 19 | 30 | 19 | 11 | 4 | 11 | NA | NA | NA | 20-60 | Below 60 | Saudi Arabia | HIC | 27.2 (10.8) | Above 10 |
| Asghar, 2024 | 396 | 296 | 396 | 296 | 100 | 75 | NA | NA | NA | NA | 48.61 | Below 60 | Pakistan | LMIC | 10.29 (6.13) | Above 10 |
| Chee, 2022 | 1774 | 470 | 317 | NA | NA | 106 | 1457 | NA | NA | 364 | 71.3 | Above 60 | Singapore | HIC | NA | NA |
| Dhasmana, 2021 | 200 | 110 | 200 | 110 | 90 | 37 | NA | NA | NA | NA | 18-59 | Below 60 | India | LMIC | 5.6 | Below 10 |
| Esubalew, 2024 | 846 | 427 | 846 | 427 | 419 | 375 | NA | NA | NA | NA | 24-70, 51.6 | Below 60 | Ethiopia | LIC | 5 (8.25) | Below 10 |
| Hlayisi, 2019 | 192 | 77 | 110 | 61 | 49 | 24 | 82 | 16 | 66 | 5 | 18-55 | Below 60 | South Africa | UMIC | 6.24 | Below 10 |
| Jordan, 2022 | 291 | 210 | 291 | 210 | 81 | 31 | NA | NA | NA | NA | 20-69 | Below 60 | United States | HIC | NA | NA |
| Kabeya, 2013 | 2753 | 592 | 483 | 182 | 354 | 129 | 2270 | 410 | 1996 | 274 | 57.8 | Below 60 | Japan | HIC | NA | NA |
| Lee, 2023 | 3168 | 1593 | 2039 | 900 | 1139 | NA | 1129 | 693 | 436 | NA | 68.92 | Above 60 | Korea | HIC | NA | NA |
| Li, 2020 | 125 | 41 | 65 | 41 | 24 | NA | 60 | NA | NA | NA | 45.8 | Below 60 | China | UMIC | 9.7 (6.0) | Below 10 |
| Naik, 2018 | 100 | 69 | 100 | 69 | 31 | 32 | NA | NA | NA | NA | 30-60 | Below 60 | India | LMIC | NA | NA |
| Pathak, 2017 | 100 | 60 | 100 | 60 | 40 | 10 | NA | NA | NA | NA | 31-55 | Below 60 | India | LMIC | 1 to 21 years | Above 10 |
| Ren, 2017 | 260 | 85 | 160 | 85 | 75 | 61 | 100 | NA | NA | NA | 50.68 | Below 60 | China | UMIC | 5 | Below 10 |
| Shaikh, 2020 | 196 | 34 | 98 | 33 | 65 | 1 | 98 | 1 | 97 | 0 | 20-40 | Below 60 | India | LMIC | NA | NA |
| Mitchell, 2009 | 1858 | 735 | 210 | 105 | 105 | 101 | 1648 | 630 | 1018 | 597 | 70.5 | Above 60 | Australia | HIC | 7.43 | Below 10 |
| Bamanie, 2011 | 196 | 110 | 109 | 76 | 33 | NA | 87 | 34 | 53 | NA | 47.9 | Below 60 | Saudi Arabia | HIC | 10.52 | Above 10 |
| Cheng, 2009_1 | 3192 | 1048 | 150 | 69 | 81 | 24 | 3042 | 979 | 2063 | 387 | 44.9 | Below 60 | United States | HIC | 4.8 | Below 10 |
| Cheng, 2009_2 | 4486 | 1038 | 391 | 183 | 208 | 76 | 4095 | 855 | 3240 | 311 | 44.3 | Below 60 | United States | HIC | 6.1 | Below 10 |
| Helzner, 2005 | 2052 | 1641 | 371 | 294 | 77 | 46 | 1681 | 1347 | 334 | 365 | 77.5 | Above 60 | United States | HIC | NA | NA |
| Mozaffari, 2010 | 160 | 52 | 80 | 36 | 44 | 22 | 80 | 16 | 64 | 7 | 45 | Below 60 | Iran | UMIC | 11.7 | Above 10 |
| Gong, 2018 | 6984 | 4110 | 388 | 279 | 109 | 129 | 6596 | 3831 | 2765 | 1554 | >=60 | Above 60 | China | UMIC | NA | NA |
| Nkosi, 2024 | 35 | 11 | 35 | 11 | 24 | 6 | NA | NA | NA | NA | 41.03 | Below 60 | South Africa | UMIC | 0-10 years | Below 10 |
| Sakuta, 2007 | 303 | 304 | 103 | 62 | 41 | 48 | 442 | 200 | 242 | 135 | 52.9 | Below 60 | Japan | HIC | NA | NA |
| Srinivas, 2016 | 50 | 33 | 50 | 33 | 17 | 6 | NA | NA | NA | NA | 31-65 | Below 60 | India | LMIC | 9.26 | Below 10 |
| Mishra, 2024 | 152 | 107 | 152 | 107 | 45 | 40 | NA | NA | NA | NA | 49 | Below 60 | India | LMIC | 10.9 | Above 10 |
| Uchida, 2010 | 2306 | 0 | 151 | NA | NA | NA | 2155 | NA | NA | NA | 40-86 | Above 60 | Japan | HIC | NA | NA |
